# Exploration of *Klebsiella pneumoniae* M6 for paclobutrazol degradation, plant growth attributes, and biocontrol action under subtropical ecosystem

**DOI:** 10.1371/journal.pone.0261338

**Published:** 2021-12-16

**Authors:** Govind Kumar, Shatrohan Lal, Shailendra K. Maurya, A. K. Bhattacherjee, Parul Chaudhary, Saurabh Gangola, Shailendra Rajan

**Affiliations:** 1 ICAR-Central Institute for Subtropical Horticulture, Lucknow, India; 2 Department of Animal Biotechnology, NDRI, Karnal, Haryana, India; 3 Graphic Era Hill University, Bhimtal, Uttarakhand, India; Babasaheb Bhimrao Ambedkar University, INDIA

## Abstract

In recent times, injudicious use of paclobutrazol (PBZ) in mango orchards deteriorates the soil quality and fertility by persistence nature and causes a serious ecosystem imbalance. In this study, a new *Klebsiella pneumoniae* strain M6 (MW228061) was isolated from mango rhizosphere and characterized as a potent plant growth promoter, biocontrol, and PBZ degrading agent. The strain M6 efficiently utilizes PBZ as carbon, energy and nitrogen source and degrades up to 98.28% (50 mgL^-1^ initial conc.) of PBZ at 15^th^ day of incubation in MS medium. In the soil system first order degradation kinetics and linear model suggested 4.5 days was the theoretical half-life (*t*_*1/2*_ value) of PBZ with strain M6. Box Behnken design (BBD) model of Response surface methodology (RSM) showed pH 7.0, 31°C temperature, and 2.0 ml inoculum size (8 x 10^9^ CFU mL^-1^) was optimized condition for maximum PBZ degradation with strain M6. Plant growth promoting attributes such as Zn, K, PO_4_ solubilization IAA, HCN and NH_3_ production of strain M6 showed positive results and were assessed quantitatively. The relation between plant growth promotion and PBZ degradation was analyzed by heat map, principal component analysis (PCA) and, clustal correlation analysis (CCA). Strain M6 was also showing a significant biocontrol activity against pathogenic fungi such as *Fusarium oxysporum* (MTCC–284), *Colletotrichum gloeosporioides* (MTCC– 2190), *Pythium aphanidermatum* (MTCC– 1024), Tropical race 1 (TR -1), and Tropical race 4 (TR -4). Hence, results of the study suggested that strain M6 can be utilized as an effective bio-agent to restore degraded land affected by persistent use of paclobutrazol.

## 1. Introduction

Paclobutrazol (PBZ) is a frequently used crop protectant in agriculture and horticulture [1]. The extensive usage of PBZ has major effects on public health and associated ecosystem. The use of PBZ has an impact on both the land and the water. Excessive use of PBZ in agriculture pollutes soil and water systems. PBZ is a plant growth hormone that stimulates and controls plant vigour in a range of annual fruit and vegetable crops, and also blooming, fruiting, and tree health. PBZ belongs to triazole group compounds and well-known for systemic fungicides action with growth-controlling effects in plants. They’re also known as stress protectants and successfully develop stress tolerance in plants by boosting their antioxidants molecules [2]. When PBZ sprayed or soaked in the soil it regulates active chemical that affects nearly all plant species. It is sprayed on perennials and other potted plants at dosages of 1–90 mg L^-1^ [3]. In seed germination studies of Arabidopsis plants, paclobutrazol resistances may alter salt and abscisic acid responses redundantly [4].

Paclobutrazol has half-life (43–618 days) and persist in the nature for long duration due to absorption/adsorption in the soil particles [5–8]. In the soil, it has a residue level of 1.1–150 mg kg^-1^ [9, 10], and has a major effect on soil microbiome [7, 11, 12] and further grown crops [7, 11]. Despite the massive volumes of PBZ used in crops and the possibility of PBZ-like chemicals contaminating groundwater [13–15], its destination and toxicity remain unknown [16]. Absorption by the plants and the production of bound residues in soils and fruits are the major problem associated with PBZ research [15, 17–19]. Till date it is unknown how PBZ interacts with soil nutrients, and enzymes; and how it affects absorption and transportation pathways during rainy season [20, 21]. In terms of PBZ stability and degradation, multiple field tests have showed that paclobutrazol has an extremely long half-life (180 to 973 days) in the environment [7, 17].

To reduce PBZ concentration from soil, bacteria such as *Pseudomonas sp*. or *Acinetobacter*, have been reported to breakdown PBZ to nearly negligible levels, with t_*1/2*_ values ranging from 13 to 95 days [6, 7]. In contrast to its growth-retarding effects on agricultural plants, paclobutrazol’s suppressive influence on microbial populations has been noted in a few research papers. Silva et al. [7] employed a conventional approach to show that paclobutrazol reduced the microbial populations in PBZ-treated Brazilian soils, confirming paclobutrazol’s antibacterial properties. Soil enzymes considered as indicator for ecological stress in both natural and agricultural systems. Soil enzymes especially dehydrogenase activity has been used to analyze microbial activity in pesticide-treated soil [6]. Dehydrogenase is an indicator of PBZ’s impact on soil microbes. Silva et al. [7] discovered a 58% reduction in total bacteria count in soil treated with paclobutrazol (160 g a.i./g) compared to unamended soil. While PBZ is widely used in mango farming to address the problem of alternate bearing, research shows that it may retain for last 6 months in field soils and has a negative impact on soil microbiome [7].

High concentration of paclobutrazol builds up suffocating environment for microbiological enrichment in the soil system [7]. For management of PBZ use and its reduction recently microbial strain *Pseudomonas putida* stain T7 reported to degrade the PBZ efficiently with plant growth promotory action under subtropical conditions [16]. Likewise, *Klebsiella* spp. despite climatic variation abundant in the subtropical ecosystem and identified as potential aromatic hydrocarbon degrader with plant growth promotory properties in different crop systems [22]. *Klebsiella pneumoniae* DNA displays versatility in surviving in many microhabitats and also showed plant growth promoting attributes. The presence of genes related in plant colonization and proliferation was discovered in *K*. *pneumoniae* 342 bacteria [23]. Based on the *Klebsiella pneumoniae* metabolic versatility and the problems associated with PBZ in environmental fate, biodegradation mechanisms of action and overall packages of practice for its effective use without detrimental effects on soil health lead to the following objectives for this study (i) identification and characterization of *Klebsiella pneumoniae* strain M6 for plant growth promotion, bio-control action, degradation potential and soil enzyme activity, (ii) By using response surface methodology optimization of PBZ degradation, (iii) strain M6 growth-linked degradation of PBZ, (iv) Biodegradation assay of PBZ and its metabolic kinetics using HPLC and GC-MS.

## 2. Materials and methods

### 2.1. Chemicals used for experimentation

Mineral salt medium (MSM) (Hi-media Laboratories) was used for enrichment and isolation of PBZ degrading bacterial culture containing (g L^-1^) (NH_4_)_2_SO_4_ 2.0, MgSO_4_.7H_2_O 0.2, CaCl_2_.2H_2_O 0.01, FeSO_4_.7H_2_O 0.001, Na_2_HPO_4_.12H_2_O 1.5, KH_2_PO_4_ 2.0, Glucose 2.5. Luria Bertani agar medium containing gram per litre NaCl 10.0 g, Tryptone 10.0 g, Yeast extract 5.0 g, Agar 15.5 g was used for culture maintenance. Potato dextrose agar (PDA) Potato infusion 200.0 g, Dextrose 20.0 g, Agar 15.5 gm L^-1^ and Rose Bengal Chloramphenicol medium having mycological Peptone 5.0 g, Dextrose 10.0 g, KH_2_PO_4_ 1.0 g, MgSO_4_.7H_2_O 0.50 g, Rose Bengal 0.05 g, Chloramphenicol 0.10 g, Agar 15.50 g were used for fungal colony growth and enumeration. Paclobutrazol (99.9%), Carbosulfan (98.0%) Imidacloprid (99.9%), and Chlorpyrifos (94.0%) were analytical grade compounds and purchased from Merck KGaA, Darmstadt, Germany, was used for experimentation purposes.

### 2.2. Rhizobacterial isolation by culture enrichment technique

Well established Mango orchards (Research Farms of ICAR-CISH Rehmankhera, Lucknow India coordinates 26°54’15.2"N 80°45’55.5"E) frequently treated with paclobutrazol were selected for soil sampling. To enrich bacterial isolates one gram soil sample from each orchard was transferred to Erlenmeyer flask (250 mL) containing 50 mL sterile liquid MSM broth. Paclobutrazol (50 mgL^-1^) from stock solution (5 gL^-1^) was maintained in each Erlenmeyer flasks. Incubate the flask into orbital shaker (REMI CIS-24 Plus) for 5 days at 30±2°C temperature and 120 rpm shaking. 1 mL of fresh culture (after acclimatization) was shifted sporadically into new sterilized flasks having liquid MSM with 10 mgL^-1^ of PBZ. Again incubate the flasks for 5 days with same parameters. Following similar process, the enhanced culture was transferred sporadically into liquid MSM medium in Erlenmeyer flasks with increasing concentrations of paclobutrazol such as 20, 40, 80, 100 and 150 mg L^-1^ over the 5 following days at 30±2°C temperature and 120 rpm shaking. Final culture from each flask spread (after dilutions) on MSM agar medium amended with 50 mg L^-1^ PBZ. Single colonies were picked and purified in triplicates on solid Luria Bertani plates after being cultured on solid minimal salt media containing 50 mgL^-1^ paclobutrazol. Further, for molecular identification 16S rRNA molecular gene sequencing technique was followed by using 27f and 1492R universal primers. After sequencing the sequences were submitted to the Gene bank NCBI, where they were assigned accession numbers. Furthermore, bacterial isolates were chosen based on their ability to degrade PBZ and plant health promotion characteristics. Among all, test strain M6 was showing the highest PBZ acclimation, degradation capacity and strong plant health attributes, therefore it was chosen for further tests.

### 2.3. Plant health attributes of selected bacterial isolate

Strain M6 was tested for different PGP characteristics such as phosphate solubilization, Zn solubilization, K solubilization, HCN and Ammonia production, IAA production, and antagonistic interaction qualitatively and quantitatively on their respective media.

#### 2.3.1. P- Solubilization

Test strain M6 was tested for Phosphate solubilization activity on solid Pikovskaya agar medium containing 0.5%, TCP (tricalcium phosphate). The strain was spotted on solid Pikovskaya agar and incubated for 96 h at 30±2°C; a clear zone around the colony indicated the positive results of the phosphate solubilization activity [24]. Further, quantification was done by following Molybdate Blue Method [25]. Briefly, liquid Pikovskaya medium was inoculated with bacterial culture in Erlenmeyer flask and incubated for 7 days at 30°C temperature on continuous shaking speed at 100rpm. Supernatant was retrieved and absorbance was recorded at 660 nm using UV Visible spectrophotometer (Model TS2080). All the tests were performed in triplicates.

#### 2.3.2. Zn solubilisation

In order to test Zn solubilization rhizobacterial isolate M6 was inoculated on tris minimal salt (TMS) agar plate containing 0.1% ZnO. The medium (TMS) was containing gram per litre D-glucose10 g, Tris HCl6.06 g, NaCl 4.68 g, KCl 1.49 g, NH_4_Cl 1.07 g, Na_2_SO_4_ 0.43 g, MgCl_2_.2H_2_O 0.2 g, CaCl_2_.2H_2_O 30 mg, and Agar 15g. Media was amended by ZnO (0.1%) as a sole source of Zn. The plates were placed for 48 h at 30±2°C and formation of halo zone around the colony was indicating the positive result of zinc solubilization activity. Further, for quantitative estimation standard method described by Mumtaz et al. [26] was followed with slight modification. In brief tris minimal salt medium having 0.1% ZnO was inoculated with freshly grown bacterial culture. Retrieved supernatant was tested for pH and 1 mL was used for zinc estimation by using atomic absorption spectrophotometer.

#### 2.3.3. Potassium solubilisation

In order to assess the K-solubilization activity the bacterial isolates were inoculated on Aleksandrov medium including gL^-1^ 5.0g glucose; 0.5g MgSO_4_.7H_2_O; 0.1g CaCO_3_; 0.006g FeCl_3_; 2.0g Ca_3_(PO_4_)_2_; 3.0g Potassium aluminium silicate; and 20.0g agar in deionized water. Plates were placed for static incubation at 30±2°C for 4 days. The halo zone around the colonies exhibits positive results of K solubilization [27]. For quantitative estimation liquid Aleksandrov medium was inoculated with 48 hour bacterial culture. 1 mL supernatant was placed into 50 mL volumetric flask, which was filled with distilled water and carefully mixed. To estimate the amount of K in the solution, an atomic absorption spectrophotometer was used. Different concentrations of KCl solution was prepared to create standard curve, by trending the standard curve, the quantity of K-solubilized by the bacterial isolates was estimated [28].

#### 2.3.4. IAA production

Rhizobacterial isolate was tested for indole–3–acetic acid production by following the standard method described by Bric et al. [29]. For quantitative estimation supernatant (2 mL) was acidified with orthophosphoric acid (two drops) and 4 mL of Salkowski reagent (1 mL of 0.5 M FeCl_3_ in 50 mL of 35% perchloric acid). Test tubes were then incubated at room temperature in dark for 25 min. The absorbance at 535 nm was recorded and interpolated against standard curve of different concentrations of IAA [30].

#### 2.3.5. HCN production

The test strain was screened for HCN production, for this LB agar media plates was prepared by amending the 4% glycine L^-1^. Sterilized Whatman filter papers soaked in 2% sodium carbonate and 0.5% picric acid solution and were placed on lid of the plate, after inoculation of bacterial isolates. The plates were wrapped properly with the help of parafilm and incubated at 30±2°C for 72 h. After incubation appearance of orange/dark brown color from yellow confirmed the positive results [31]. For quantitative estimation standard method described by Kremer and Souissi [32], was followed. Briefly NaOH (5.0 mL 1.0 M) extracted cyanide was titrated against 4.25 mL acetic acid. Extracted cyanide was reacted with barbituric acid-pyridine reagent and absorbance was recorded at 575nm with the help of spectrophotometer.

#### 2.3.6. Ammonia production

Ammonia production was checked according to Cappuccino and Sherman [33]. Briefly, rhizobacterial isolate was grown in peptone water broth for 72 hours at 30±2°C and 100 rpm shaking. The supernatant (5 mL) was mixed with 2 mL Nessler’s reagent; appearance of yellow to brown colour indicates the positive result for NH_3_ production.

#### 2.3.7. Antagonistic interaction with strain M6 against selected plant pathogens

Dual-culture plate and relative fungal pathogen growth approach were performed for antagonistic interaction experiment. Freshly grown fungal pathogens were interacted with bacterial isolates placed at same plate at the both side of the plate. Plate only with fungal pathogen was considered as control. All test plates were placed for 7 days incubation at 28±2°C. All experiments were done in triplicate. The percent suppression of pathogen mycelium growth over control was estimated by measuring the radial growth of fungal mycelium according to formula below–

$$\%Inhibition=\frac{\left(C-T\right)}{C}\times 100$$

Where ‘C’ is the fungal mycelium colony radial growth (in mm) of control and ‘T’ is the radial growth (in mm) of the fungal mycelium growing in dual inoculation with antagonizing test strain.

### 2.4. Bacterial growth-connected degradation of PBZ by strain M6

The bacterial growth connected PBZ degradation test was done with strain M6. Active culture (12h) of strain M6 was transferred to 500 mL liquid MSM media amended with 150 mg L^-1^ PBZ. Flask without bacterial culture was considered as a control. UV/Visible spectrophotometer was employed to measure bacterial growth at 600 nm. From each flask’s residual paclobutrazol was extracted at definite time interval (3, 6, 9, 12 and, 15 days) and evaluated using high-performance liquid chromatography (HPLC). For bacterium M6, growth-linked degradation of bacterial cell density and paclobutrazol degradation was assessed.

### 2.5. Kinetic study of PBZ degradation with test strain M6

Single First order degradation kinetics model was applied for each treatment and calculated accordingly. Along with PBZ three other pesticides (imidacloprid chlorpyrifos and carbosulfan) were tested for the biodegradation experiment with strain M6. Three replicates of each experiment were evaluated, along with control. At definite time intervals (7, 15, and 30 days after inoculation), samples were withdrawn and extracted for residual pesticide in soil, and HPLC analysis was performed. To test the pesticide degradation, soil was collected from top layer (0–30 cm). The soil was taken from field that had never been treated with pesticides before. The soil was wet sterilized for 1 hour at 121°C for three consecutive days. The soil (500 g) was spiked with PBZ at 50mg L^-1^ under sterilized and natural soil conditions. The use of this aqueous solution helped to keep the soil wet. All the tests were carried in sterile plastic pots. The young grown bacterial population was adjusted to 1x10^-8^ after centrifugation. Under sterilized environment, this culture with a comparable optical density was combined with all of the soil treatments. In triplicates, uninoculated sterile and natural soil samples lacking bacterial culture served as control. 10 gm of soil sample from each treatment were collected and extracted for PBZ and other pesticides and quantified by using HPLC and GC-MS.

### 2.6. Optimization of PBZ biodegradation

To attain utmost PBZ degradation with strain M6 environmental parameters (pH, Temp, and inoculum size) were optimized in controlled condition. To optimize the variables, the Box–Behnken design (BBD) was applied, which was based on Response surface methodology (RSM) algorithm. The variables pH, Temp., and Inoculum size were optimized and tagged as X1, X2, and X3 respectively. Under controlled laboratory conditions, 17 experimental runs with 17 distinct assemblages of three components were executed. A second-order polynomial function was fitted to connect the relation between independent factors and response by anticipating the optimal point. Below is the fitted equation for the three components.

$$Y_{pred}=\beta_{{}^{\circ}}+\sum \beta_{i}X_{i}+\sum \beta_{ii}X_{i}^{2}+\sum \beta_{ij}X_{i}X_{j}$$ (1)

where Y_*pred*_ is the response predicted (for % PBZ degradation), x_*i*_ and x_*j*_ are variables in the input which influence the response Y. β_o_ is the constant, β_*i*_ is the *i*th linear coefficient, β_*ii*_ is the *i*th quadratic coefficient, and β_*ij*_ is the *ij*th interaction coefficient. To fit the data, a quadratic polynomial which includes all interactions was used. The statistical experimental design was created and evaluated by using the “Design-Expert” program (version 12, Stat-Ease Inc., Minneapolis, USA). The optimal essential condition for PBZ degradation was discovered by elaborating the regression equation (Eq 1) and examining response surface contour plots [34].

### 2.7. Extraction of PBZ

The residual paclobutrazol was extracted by using methanol. At predefined time intervals, 10 gram of soil sample was collected from each treatment and transferred into 50 mL screw capped Erlenmeyer flasks. In each flask 50 mL methanol was poured and the contents were shaken constantly for 5 hours. Cooled methanol (10 mL) was added to the samples again, and they were shaken constantly for one hour. The residual paclobutrazol were separated through Whatman no 4. filter paper and collected into new 25mL beaker. For complete recovery, again 10 mL methanol was added in flask and proceeded further. Using a vacuum evaporator, the surplus solvent was removed from the samples. The totally evaporated samples were processed to recover paclobutrazol using methanol (HPLC grade) and subjected to HPLC (Model-Nexer-R, SIL-30ACMP, Shimadzu Japan) analysis fitted with a C18 column. Under vacuum pressure, deionized water and filtered (0.45 m filter) methanol were utilized as the mobile phase. The mobile phase ratio was set at 65 percent methanol and 35 percent water, with a flow rate of 0.7 mL min^-1^ and isocratic pumping. After injecting a 25μL sample, UV detection was performed at 227 nm with 7.15 minute retention duration. Gas chromatography-mass spectrometry (GC–MS) (Model-TQ 8050 Nexis Shimadzu Japan) analysis was done for the detection of intermediate compounds during paclobutrazol degradation at AIRF facility of JNU, New Delhi, INDIA. Using the National Institute of Standards and Technology (NIST) library database, the degradation metabolites of paclobutrazol were identified and compared. The intermediate metabolites of paclobutrazol degradation were identified using the NIST collection based on their retention time (RT) and molecular weight (m/z).

### 2.8. Statistical analysis

For fitting biodegradation kinetics statistical analysis software package, (Origin Pro 2018, MA, USA) was used while “Design-Expert” software (version 12, Stat-Ease Inc., Minneapolis, USA) was used for optimization purpose to plot the 3D response surface plot with a level of significance of 95% (p = 0.01 and 0.05). A one-way analysis of variance (ANOVA) was used to verify the data, and the DMRT test was used to compare the means.

## 3. Results

### 3.1. Isolation of PBZ utilizing bacteria

**Fig 1:**
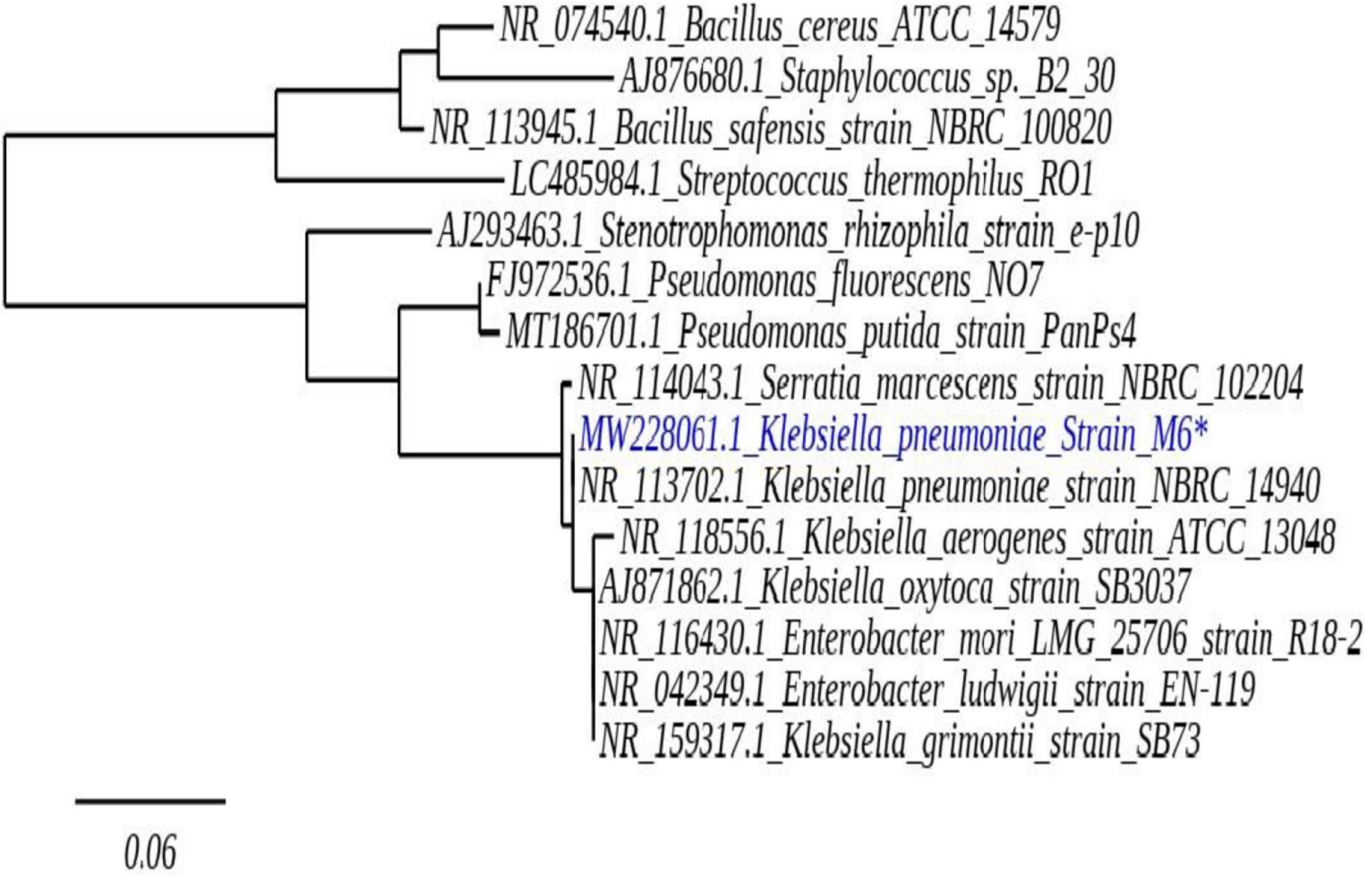
Phylogenetic relationship of strain M6 inferred by neighbor-joining method. Evolutionary analysis was conducted by online tool phylogeny (phylogeny.fr/simple_phylogeny.cgi).

Total twelve morphologically distinct rhizobacterial isolates were isolated from twelve samples collected from mango orchard rhizosphere soils. The soil has previously reported for regular PBZ application under subtropical condition [35, 36]. For culturable microbial diversity samples (100μl) were spread on four different agar (Nutrient agar, Potato dextrose agar, Rose Bengal chloramphenicol agar, Actinomycetes isolation agar) plates containing (50mg L^-1^) PBZ. Results showed that sample S2 was showing highest CFU count followed by sample S7, S6, S8, S9, S10, S1, S3, S4, S5, S11 and S12 (S1 Fig). Further, twelve morphologically distinct bacteria (M1 to M12) were showing high potential towards PBZ were isolated from the total samples, all of which using PBZ (50–150 mg L^-1^) as their only carbon, nitrogen, and energy source. Out of twelve, isolate M6 was showing great PBZ degradation (estimated by HPLC) and better stability in PBZ amended media. Further, based on comparative analysis of 16S rRNA sequences it was found that isolate M6 was closely related to *Klebsiella pneumoniae*. Moreover, for evolutionary relationship a phylogenetic tree was constructed by using neighbour joining method (Fig 1). The sequences obtained, were submitted to NCBI data base under accession no. MW228061.

### 3.2. Plant health attributes by strain M6

**Fig 2:**
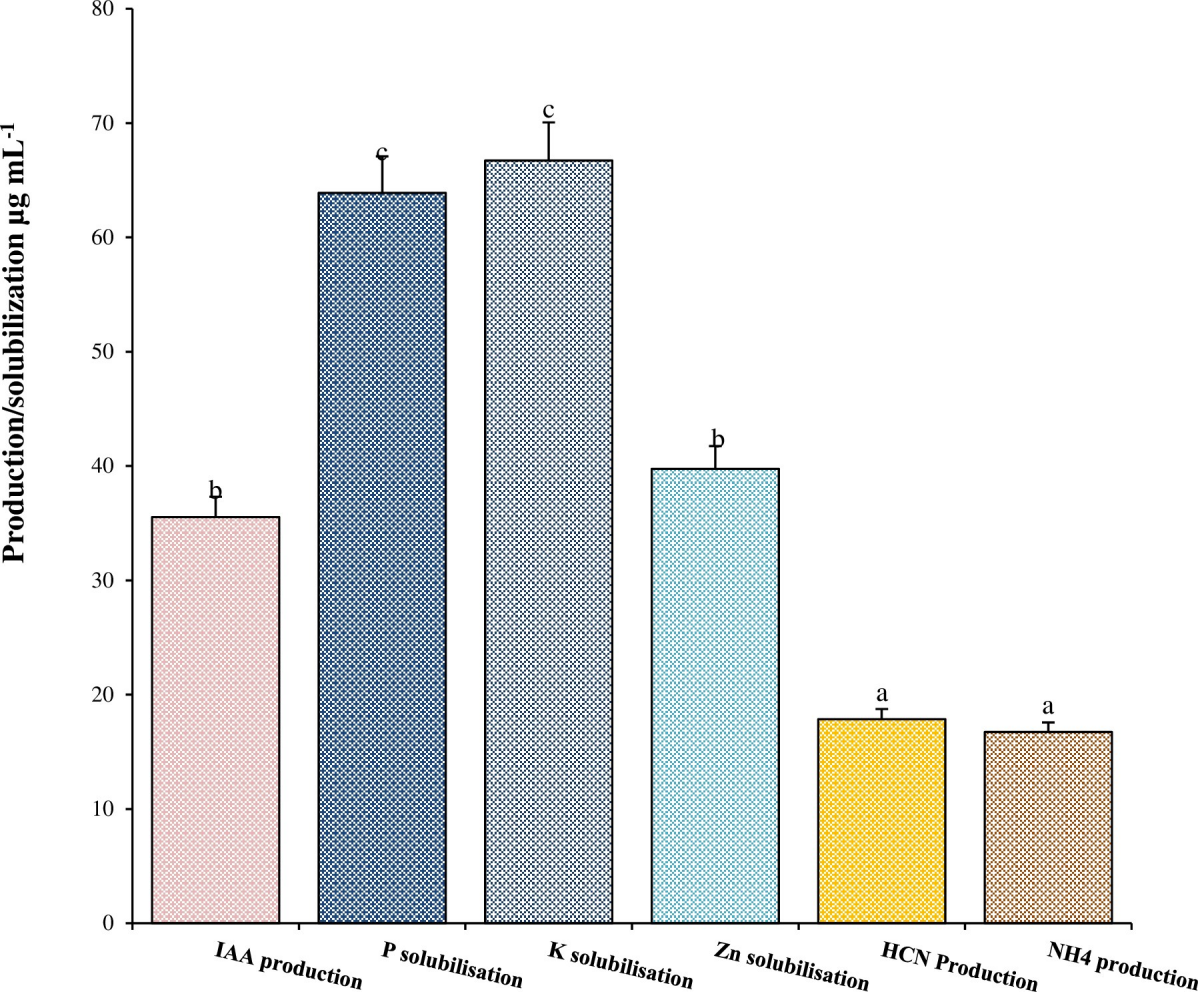
Plant growth promoting activities of the strain M6.

It was observed that strain M6 was showing multiple PGP characteristics on their respective media. The obtained results showed that strain M6 exhibited positive result for phosphate solubilization, zinc solubilization, and potassium solubilization while produced enough amount of IAA by utilizing L-tryptophan. Strain M6 was also shown positive test for HCN, and Ammonia characteristics which are well reported indirect plant growth promoting characteristics. Further quantitative estimation of PGP characteristics revealed that strain M6 produced 35.53±0.2 μgmL^-1^ of IAA in TSB medium amended with 0.5% L-tryptophan while produced 17.85 ±0.13 and 16.73±0.11 μgmL^-1^ of HCN and Ammonia respectively in their respective medium. Moreover, strain M6 solubilized 39.75±0.12, 63.89±0.27, and 66.72±0.22 μg mL^-1^ of macro and micro essential elements zinc, phosphate, and potassium, respectively (Fig 2).

### 3.3. Antagonistic interaction between strain M6 and various plant pathogens

**Fig 3:**
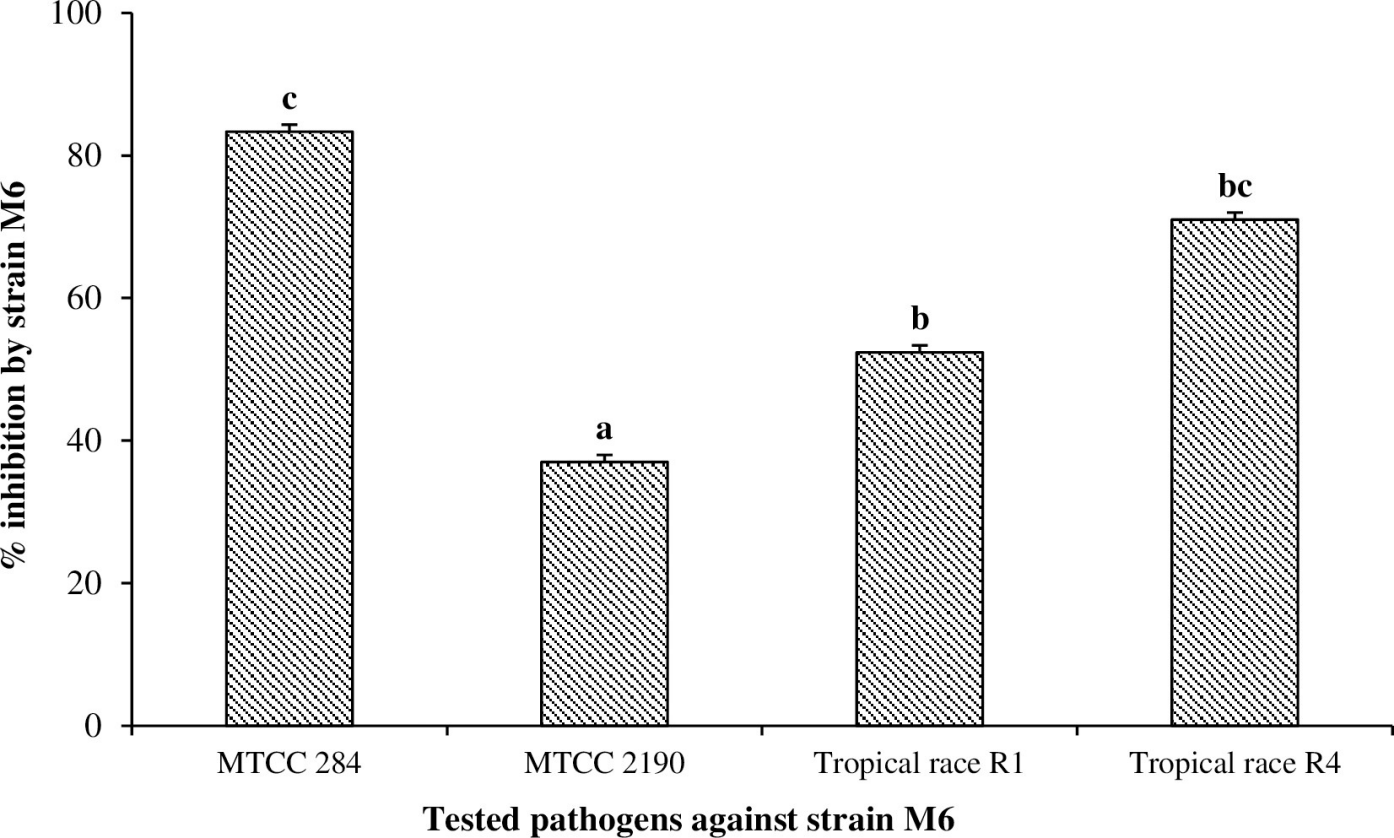
Biocontrol action of strain M6 against different plant pathogens.

*In-vitro* investigation of antagonistic interaction with strain M6 against four fungal phytopathogens revealed that, strain M6 showed strong antagonistic interaction against *Fusarium oxysporum* (MTCC-284) with 83.33±0.11% inhibition, *Colletotrichum gloeosporioides* (MTCC– 2190) with 37.00±0.11% inhibition, *Tropical race* R1 with 52.37±0.12% inhibition and *Tropical race* R4 with 71.00±0.12% inhibition while no inhibition was observed against *Fusarium oxysporum* (MTCC-10247) by strain M6 (Fig 3).

### 3.4. PBZ degradation optimization by Response Surface Methodology (RSM) using Box-Behnken Design (BBD)

To find the optimum condition for maximum PBZ biodegradation, experiments were performed with the help of RSM using three factorial Box-Behnken Design. Different combinations of three factors pH (X1), Temperature (X2) and Inoculum size (X3) total 17 experiments were conducted. To compare between responses and variables second order polynomial equation was followed. A predicted function Y (% PBZ degradation) was obtained by fitting the response (R1) data in quadratic model–

$$Y=+90.89783+1.33938X_{1}+0.010222X_{2}+0.006389X_{3}‐0.000083X_{1}X_{2}+0.000667X_{1}X_{3}‐0.000111X_{2}X_{3}‐0.094750X_{1}{}^{2}‐0.000167X_{2}{}^{2}‐0.003111X_{3}{}^{2}$$ (2)

**Fig 4:**
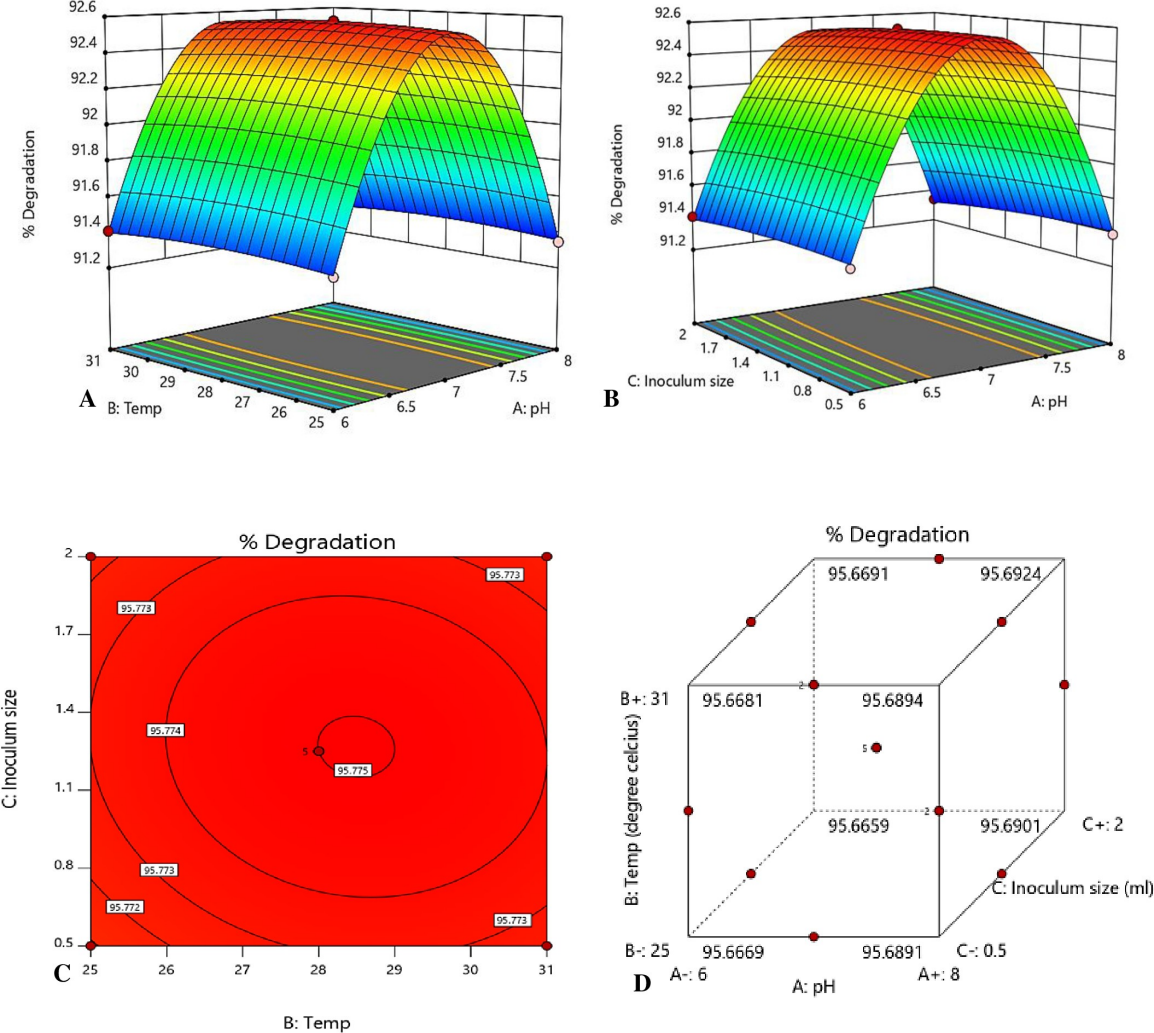
(A, B) 3D response surface plot (C) contour plot (D) cube plot of PBZ biodegradation process optimization in MS medium by strain M6.

Where, Y is predicted function for % PBZ biodegradation by strain M6 X_1_, X_2_ and X_3_ are the coded factors for pH, Temp, and Inoculum size, respectively and R1 represents the % PBZ biodegradation by strain M6. Results obtained from quadratic model were showed by 3D response surface plot, contour and cube plot (Fig 4A–4D) which represents the optimum value for highest PBZ biodegradation by bacterium M6. Thus, the quadric model results revealed that optimum condition for highest PBZ biodegradation was found at 2 mL inoculum size, 31°C temperature, and pH 7. Further, for significance level of quadric model results, analysis of variance (ANOVA) was performed at level of significance (p≤ 0.001). The coefficient determinant (R^2^) value (0.990) showed that the model’s predicted values were close to the observed values which were showing best match between the predicted and observed experimental results. The model’s high F value (17435.56) and low P value (0.001) indicated that the model was significant. Due to noise, there was only a 0.01 percent chance that an F-value of model would occur. Low P-values (≤0.05) indicated model terms were at significant level. The model components pH, Temperature, pH^2^, Temp^2^, and Inoculum size^2^ were all important in this scenario. The model terms were not significant if the value recorded more than 0.1000. The results revealed that there were relatively few inconsequential model terms (excluding those necessary to sustain hierarchy), suggesting that their removal might improve the quadric model.

### 3.5. Kinetics of PBZ degradation

The degradation constant (k), half-life (t_*1/2*_), and regression constant (R^2^) for PBZ degradation with strain M6 were determined using first order degradation kinetics and linear model. PBZ’s theoretical half-life (t_*1/2*_) value was determined using standard equation and is shown in S2 Fig. (). The theoretical half-life (*t*_*1/2*_) value of PBZ degradation with strain M6 was tested 4.05 day^-1^ in batch culture while in soil it was near to 13.99 day^-1^ with strain M6. Along with PBZ, strain M6 was also tested for degradation of three other pesticides such as carbosulfan imidacloprid, and chlorpyrifos and it was noted that strain M6 was also capable to degrade carbosulfan up to 96.29% imidacloprid up to 97.03% and 98.49% chlorpyrifos at 15^th^ day after incubation in bath culture while 30^th^ day after incubation in the soil.

### 3.6. Growth linked biodegradation of PBZ by strain M6

**Fig 5:**
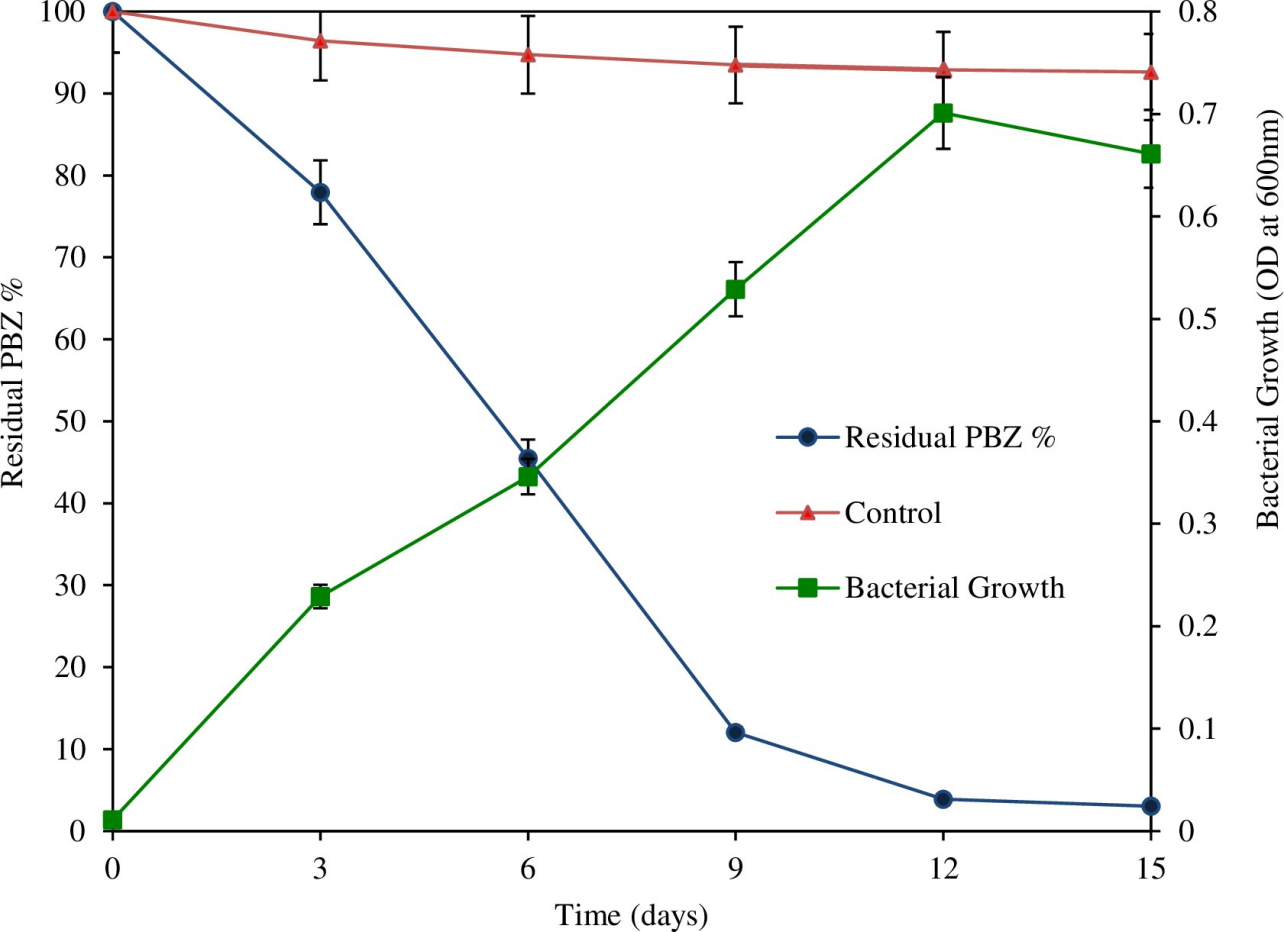
Growth-connected degradation of PBZ by strain M6.

Results from this experiment showed that growth of strain M6 increased with decreased in residual concentration of PBZ linearly i.e., degradation is directly proportional to the growth of strain M6 (Fig 5). In addition to this, it was noted that the specific growth rate (*μmax* h^-1^) of strain M6 and specific degradation rate (*qmax* h^-1^) was higher at lower concentration of PBZ while a decrease in specific growth rate and specific degradation at higher concentration of PBZ was observed. It was also noted that a prolonged lag phase (λ) showed by strain M6 under higher concentration of PBZ while, shorten lag phase (λ) recorded at lower concentration of PBZ in batch experiment. Higher concentration-effect of PBZ has also affected the specific degradation rate and bacterial growth rate i.e., at higher concentration of PBZ reduced specific degradation rate and specific growth rate. Thus, obtained results were clearly indicating that the growth of strain M6 was directly depended on the concentration of PBZ in batch culture.

### 3.7. PBZ biodegradation in different soil system and in MSM broth treated with bacterium M6

**Fig 6:**
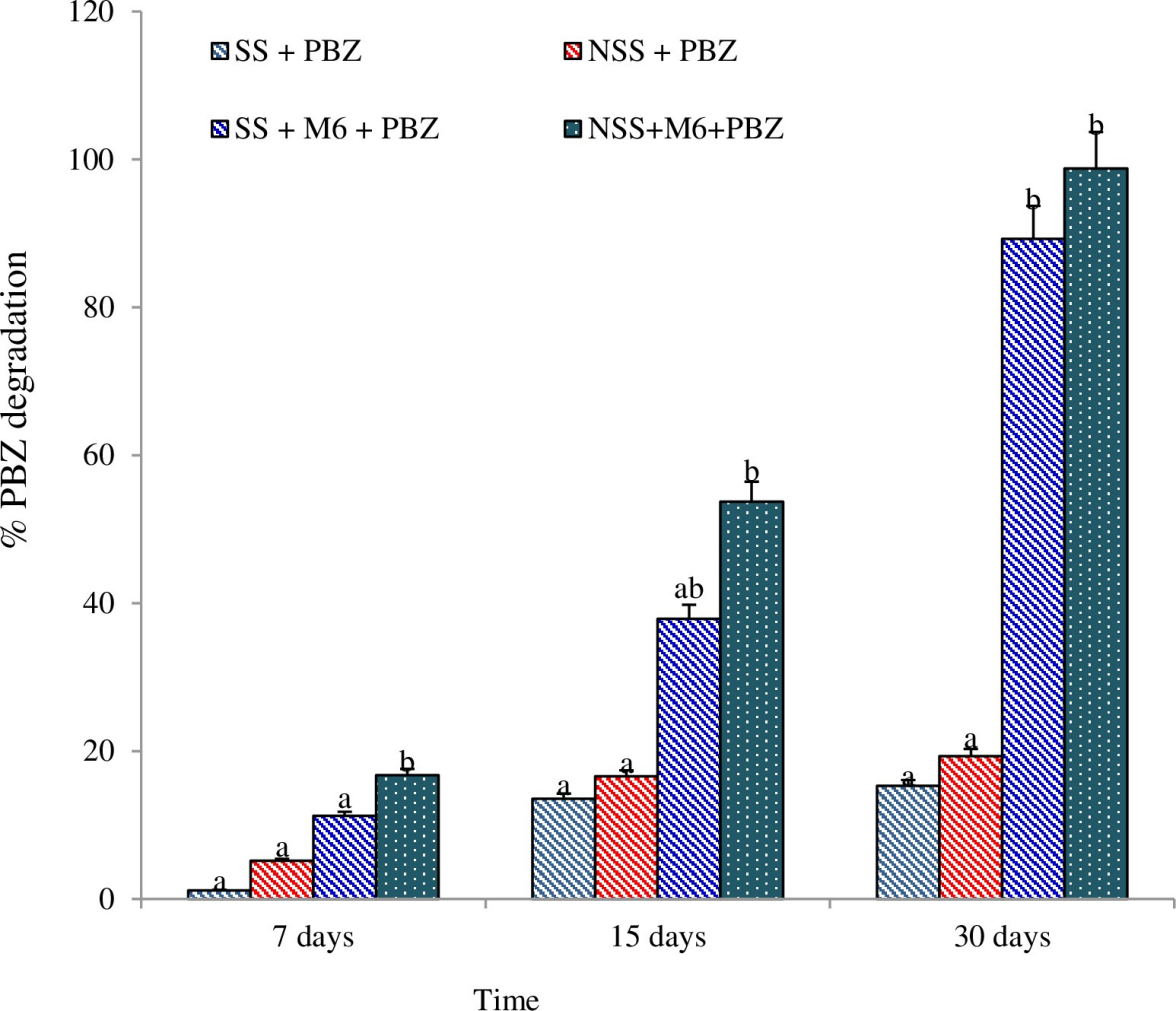
PBZ degradation in sterile and non-sterile soils with strain M6 in three different sampling.

Results from this experiment revealed that strain M6 was capable to degrade PBZ very efficiently in the soil system. It was noted that when strain M6 augmented with natural soil 16.75% degradation was observed in 7 days, however, when strain M6 augmented with sterile soil 11.22% degradation was observed in 7 day after incubation. Further, at 15^th^ day of incubation the 37.89 and 53.73% degradation was observed in sterile and natural soil, respectively, with strain M6. Moreover, at 30^th^ day of incubation 98.77% degradation was observed in natural soil while, 89.25% degradation was observed in sterile soil inoculated with strain M6 (Fig 6).

### 3.8. Soil dehydrogenase under PBZ stress

**Fig 7:**
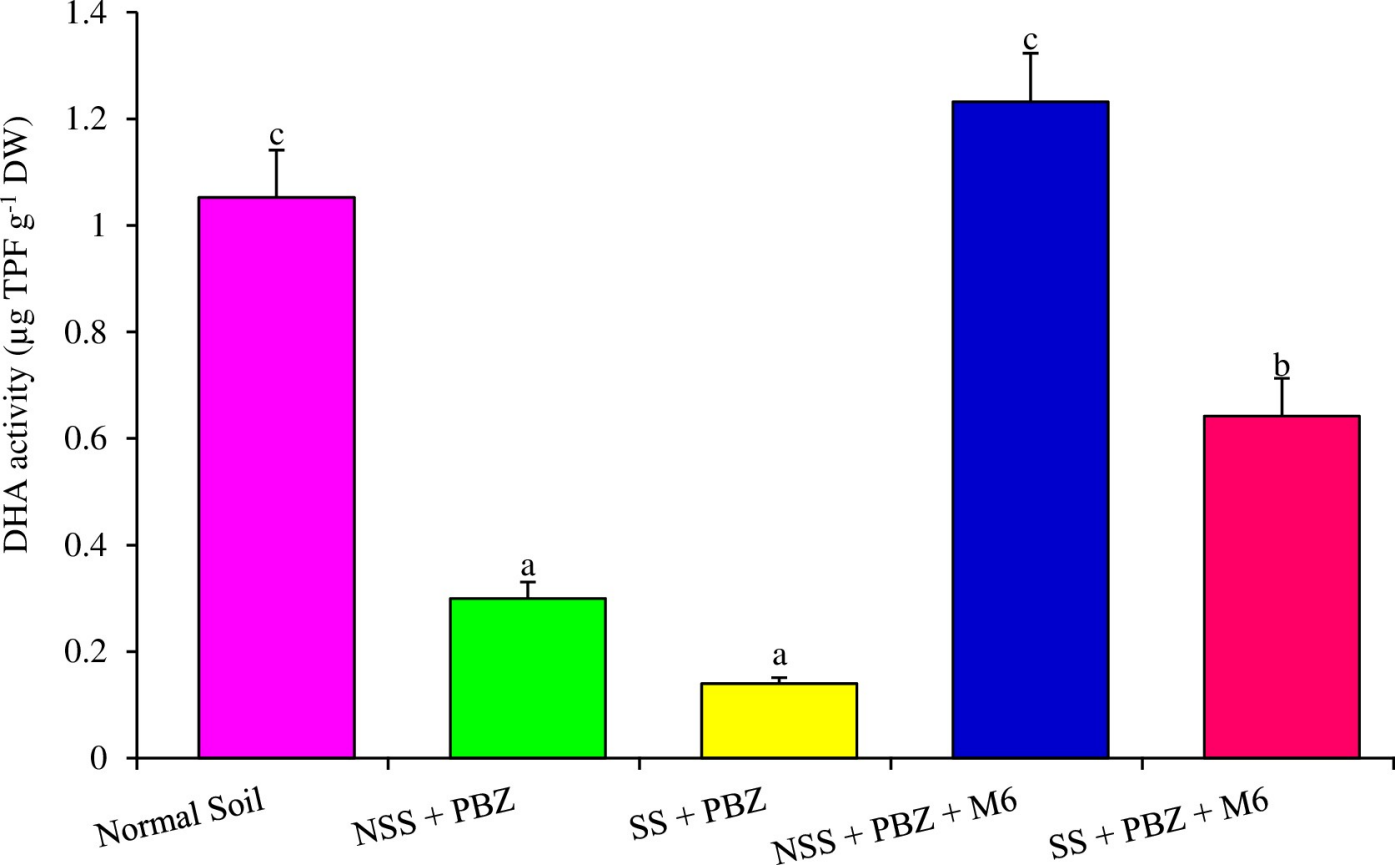
Soil dehydrogenase activity treated with PBZ (NSS-non-sterile soil, SS-sterile soil).

Soil dehydrogenase activity was assessed in PBZ contaminated soil and M6 treated PBZ contaminated soil. Results from this assay indicated that soil DHA activity was decreased by 71% in contaminated soils as compared to normal non-contaminated soils. It was also noted that DHA activity was significantly reduced in systematically sterilized soil and PBZ contamination suggested that PBZ reduced the soil microbial activity. On the other hand in natural soil and PBZ contaminated soil DHA activity was decreased indicating the toxicity of PBZ in natural soil too. Whereas, soil treated with strain M6 showing an increased DHA activity in sterile as well as natural soils (75%), suggesting that strain M6 having an enzyme system that efficiently utilized PBZ as carbon and energy source resultant decrease in soil toxicity and increase microbial activity in the soil. From Fig 7 it is well depicted that strain M6 reduced the PBZ toxicity and increases microbial activity by increasing the DHA activity.

### 3.9. Clustal correlation analysis of PBZ degradation at different time intervals

A Clustal correlation analysis based on Pearson analysis was performed to find out the linkage between PBZ degradation and incubation period by strain M6 under different treatments in the soil. Results showed that PBZ degradation was linearly positive correlated with incubation period. The correlation coefficient for percent PBZ degradation for strain M6 was 0.98 at p≤0.01. It was also observed that incubation period between 6–9 days was well correlated with degradation percent and at this the maximum degradation was achieved by strain M6. Clustal correlation by heat map analysis revealed that there were three group of incubation period i.e., 3,6,9 days was nearest to correlation coefficient (R^2^–0.99) and were showing the maximum PBZ degradation (S3 Fig).

### 3.10. Principal component analysis

**Fig 8:**
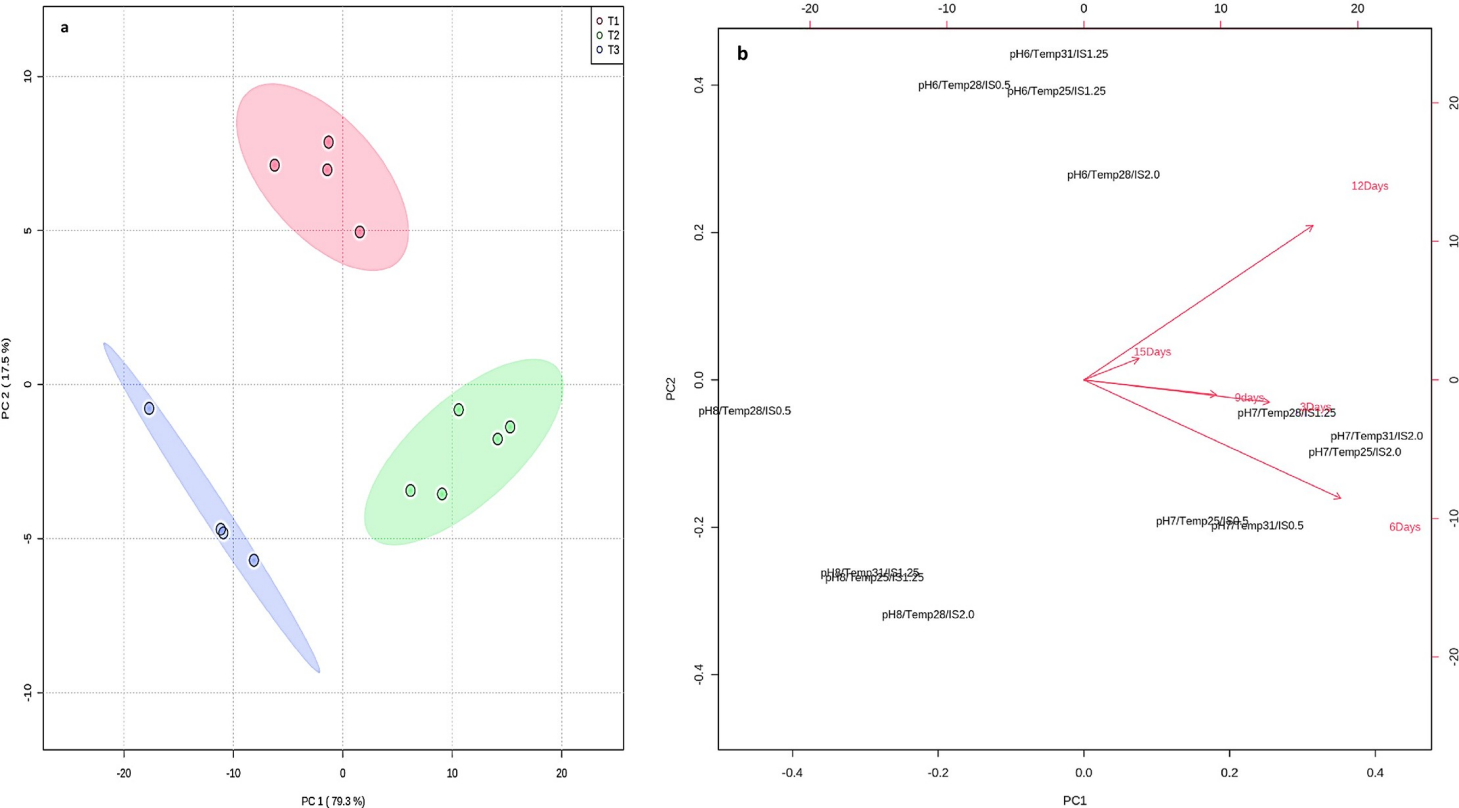
a, b. Principal component analysis of PBZ degradation by strain M6 under different time intervals. Score (a) and Loading plot (b) of different parameters analysed three treatments i.e. pH, Temp, inoculum size, under PBZ contamination. The x-axis indicates principal component 1 (PC1) and the y-axis indicates principal component 2 (PC2).

Principal component analysis (PCA) was performed for comparative analysis of PBZ degradation and three different treatment combinations (pH, Temp and inoculum size) under five different incubation periods by strain M6. The total variables of PCA were the percentage of different factors upon which the degradation of PBZ depends. The results of PCA yielded five components that explained 100% of the total variance in the data and first two components had Eigen value 1 (which is showing significance of the data), and together they describe 100% of the variance of the data obtained by PBZ degradation. From score plot and vector diagram it was cleared that variables such as 7 days (incubation period) and pH-7, Temp-28, IS-1.25 mL^-1^ were positively correlated with component one and component two. In addition to this, 15 days with same variables were positively correlated with component one and two also, suggesting that maximum PBZ biodegradation was achieved from 7 to 15 days (57 to 95%). Further, variables pH-6, Temp-28 and IS-1.25 were showed neutral correlation with component one and two, whereas variables pH -8, Temp 28, and IS 0.5 were negatively correlated with component one and two. From results, it was cleared that pH played a significant role in PBZ degradation as evident by Fig 8.

### 3.11. Hierarchical cluster analysis of PBZ degradation

To envisage dissimilarities between the treatments for PBZ biodegradation by strain M6, the outlines were separately classified by hierarchical cluster algorithm analysis based on Euclidean dissimilarity index by using Ward’s method for minimizing the information loss on merging clusters [37]. The clustering was performed by Hellinger-transformed treatment datasets, distance between the treatments was computed based on Hellinger distance. Three components *viz*. Inoculum Size, Temperature, and pH, were the main factors that were influencing the PBZ biodegradation. Hierarchical cluster analysis of PBZ biodegradation revealed 4 different clusters, (from all three treatments based on three variables i.e., pH, Temp, IS) and all the clusters were distantly correlated with each other. The first cluster is mainly based on alkaline pH 8, whereas the second cluster was based on acidic pH 6. Moreover, cluster 3 and 4 were based on neutral pH 7. It was noted that, cluster one was closely related with cluster two. Moreover, cluster three and four were closely related with each other whereas, distantly related with cluster one and two. It was evident that the relative prevalence of three factors associated with cluster 3 and 4 at which the maximum PBZ degradation was achieved. It was also observed that cluster 3 and 4 was very closely related to each other and define significantly of all three parameters i.e., pH, Temp and IS. This indicates that alkaline medium (pH– 8) showed lowest degradation whereas, neutral medium (pH– 7) showed maximum degradation of PBZ. In addition, acidic pH (pH– 6) was showing neutral correlation for PBZ biodegradation by strain M6 (S4 Fig).

## 4. Discussion

Every country’s core agricultural goal is to provide superior, nutritious, and affordable food to an ever-increasing population. Pesticides are widely applied as crop protective agents to counteract pest and disease-related losses. The negative consequences of these pesticides on human health and the environment are well documented. Researchers have discovered that microbial pesticide degradation can improve soil fertility, agricultural output, and environmental sustainability during the last few decades. Plenty of literature has already shown that bacteria having the capability to metabolize poisonous substances into non-poisonous or less toxic substances by various mechanisms. Paclobutrazol is a vegetative growth regulator that stays active in the soil and affect negatively to growth and production of subsequent crops [36, 38]. In this study, total twelve morphologically distinct rhizobacterial strains were screened and based on PBZ degradation and plant growth promotion from twelve different samples collected from mango orchards rhizosphere soil under subtropical climate (S1 Fig). Among all rhizobacterial isolates one strain *Klebsiella pneumoniae* M6 was showing maximum PBZ degradation, good PGP attributes and biocontrol action. Strain M6 was able to utilize PBZ up to 150 mg L^-1^ as carbon and energy source in batch experiment. In addition to this strain M6 was also showed a great potential to promote plant growth by direct and indirect mechanisms of PGPR. In the previous study Bhattacherjee and Singh [36], reported a longer persistence nature of PBZ (300 days) in the mango orchard soils under subtropical climate. Another study carried by Wang et al. [39], showed *Klebsiella variicola* P5 and *Klebsiella pneumoniae* P9 a good plant growth promoter and cellulose degrading agents. Similarly, Chen et al. [38], reported that a single bacterium *Pseudomonas* sp. utilize PBZ upto 60% at 48 hours of incubation in batch experiments whereas, in this study *Klebsiella pneumoniae* M6 utilized PBZ up to 98.279% in the batch experiment and showed good PGP activity which is noteworthy in this regard.

It is well reported that pesticide degrading microorganisms have the ability to promote plant growth by direct and in-direct ways [40–42]. Li et al. [40] reported that *Klebsiella* sp. PD3 was efficiently degrade phenanthrene (PHE) and showing ACC deaminase, IAA, and nitrogen fixation activity. Another study carried by Phugare et al. [43], reported *Klebsiella pneumoniae* strain BCH1 degrade imidacloprid up to 78% after 7 days at 30° C under static conditions. In this study strain M6 having the ability to degrade PBZ and demonstrate multiple plant growth attributes such as P- solubilization, zinc solubilization, potassium solubilization, HCN, ammonia production and good amount of IAA by utilizing L- tryptophan. It is clear that the selected strain M6 could survive and sustain plant growth-promoting features even in the presence of excessive paclobutrazol toxicity, enabling for decent plant growth. Moreover, during the observation of this study, it was found that there is very limited literature available in the public domain regarding pesticide degradation and plant growth promotion by the genus *Klebsiella* in a single study. This is the first report for the biodegradation of paclobutrazol (fate of PBZ) and plant growth promotion in a single study by bacterium *Klebsiella pneumoniae*.

**Fig 9:**
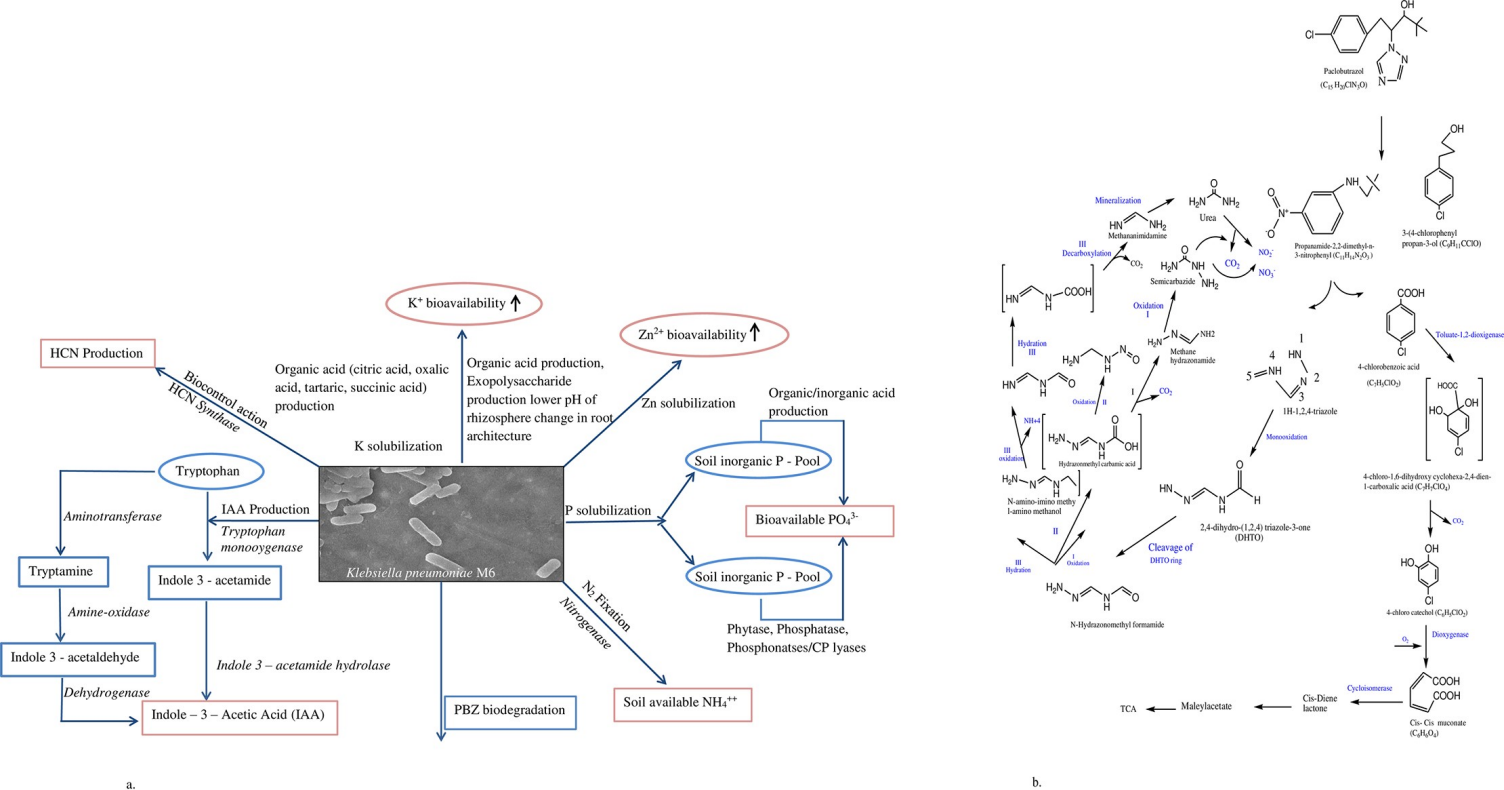
a. Mechanisms and pathways of different plant growth promoting traits by *Klebsiella pneumoniae* M6. b. Purposed biodegradation pathway of paclobutrazol by *Klebsiella pneumoniae* M6.

In a system, it is completely obvious that microbes are in usage strongly affected by several environmental factors such as temperature, pH, carbon sources, substrate concentration, nutrients, and other microorganisms present in the system. In this study response surface methodology was used for optimizing the environmental factors such as pH, temperature and inoculum size. Strain M6 was optimized with BBD design of RSM to overcome these challenges during PBZ biodegradation. Response Surface Methodology has extensively used by previous researchers to optimize process for pesticide degradation. Bhatt et al. [44, 45] and Zhan et al. [46] well applied BBD design of RSM for pyrethroids permethrin, cypermethrin, cyhalothrin, and allethrin degradation by using various soil and water source bacterial strains. The interactions of the three parameters by using Box-Behnken design of RSM for pesticide degradation well shown by this study. Study also observed that RSM was very useful for the environmental adaptability of three parameters during paclobutrazol degradation and occurs under a broad range of concentrations (50–150 mg L^-1^). The findings of this study are consistent with pyrethroid degradation studies [45, 47]. Based on the findings, the environmental inference of *Klebsiella pneumoniae* strain M6 with PBZ revealed that *Klebsiella spp*. were prevalent in mango orchards highly treated with PBZ in a subtropical habitat, Since *Klebsiella pneumoniae* has been identified as a potential PGPR and biocontrol agent (Figs 2 & 3), we investigated the M6 strain for PBZ degradation and discovered that it has a high potential for PBZ biodegradation. Every bacterium in any environment requires ideal pH, temperature, moisture and nutrient for proper growth, metabolic activity, and survival [48, 49]. In MS medium, the M6 strain followed first order kinetics for PBZ degradation and demonstrated maximal PBZ degradation of 98.29 percent. Based on PBZ degradation at various pH, temperature, and inoculum size, strain M6 displayed four clusters (1, 2, 3, and 4). PBZ degradation ranged from 91 to 98% in all clusters, although cluster 4 (pH7/temp.31°C/IS2.0) had the best results with a degradation up to 98.29%. We have observed the metabolic versatility of *Klebsiella pneuminae* by using GC-MS technique and found intermediated compounds during the breakdown of PBZ and hypothesized a metabolic route with plant growth promotory activity based on these data, the environmental fate of PBZ degradation with M6 strain (Fig 9A and 9B). In this pathway, strain M6 used PBZ effectively in this route to give nutrients to the plant system from nitrogenous part of the PBZ and maintain soil fertility in a long-term manner [16]. Paclobutrazol was degraded in such a way that the obtained intermediate compounds such as Benzene, 1,3-bis (1,1-dimethylethyl), Dodecane, 4,6-dimethyl, Heneicosane and Propanamide, 2,2-dimethyl-n-(3-nitrophenyl) and 3-(4-chlorophenyl propan-3-ol) were effectively metabolised with the help for degrading enzymes (*diooxygenase & isomerases*) and hydration & oxidation process. Finally, all intermediate compounds enter into the TCA cycle for complete utilization, and the nitrogenous component of PBZ mineralizes and becomes accessible as a nutrient for the system.

## 5. Conclusion

In this study rhizobacterial strain *Klebsiella pneumoniae* M6 was isolated from rhizospheric soil sample from PBZ contamination sites. The bacterium showing a great potential towards PBZ biodegradation (98.28%) as well as plant growth attributes by solubilizing inorganic metals such as phosphorous, potassium, and Zinc and also produced a good amount of IAA, HCN and ammonia through various ways. RSM has optimized the environmental conditions for maximum PBZ degradation. This study has also conclusive remarks that enrichment acclimation of strain M6 from contaminated soil to chronic exposure of paclobutrazol was relatively great for PBZ degradation and it was proved by principal component analysis and clustal correlation analysis. Test strain M6 proved to degrade (>50% PBZ) within 6 days. Alternatively, the half-life of paclobutrazol treated by strain M6 was approximately 9 days. By Hierarchical cluster analysis it was identified that a group of cluster (group of various factor) proved the maximum degradation achieved by RSM. Since bioremediation approaches should be chosen based on both pollutant removal efficacy and environmental consequences, in this work, indigenous bacterial strain M6 was found to have a high potential for paclobutrazol degrading plant growth promotion and bio-control action, which might aid in the development of bioremediation strategies for paclobutrazol polluted sediment or soil environments in the future.

## Supporting information

S1 Fig The Log10 CFU mL^-1^ of selected samples showing growth under 50mg L^-1^ PBZ amended MSM agar plates. (DOCX)

S2 Fig Linear model of PBZ degradation by strain M6. (DOCX)

S3 Fig Clustal pearson correlation analysis of PBZ degradation under different treatment and different time intervals. (DOCX)

S4 Fig Hierarchical cluster analysis of PBZ degradation under different treatments. (DOCX)
