# Supplementary material for: Exploration of Klebsiella pneumoniae M6 for paclobutrazol degradation, plant growth attributes, and biocontrol action under subtropical ecosystem
Source: PLoS One. 2021 Dec 16;16(12):e0261338. doi: 10.1371/journal.pone.0261338 (PMC8675670; doi:10.1371/journal.pone.0261338)
Supplement: S1 Fig — (DOCX) [file pone.0261338.s001.docx]

S1 Fig. The Log_10_ CFU mL^-1^ of selected samples showing growth under 50mg L^-1^ PBZ amended MSM agar plates.
