# Supplementary material for: Exploration of Klebsiella pneumoniae M6 for paclobutrazol degradation, plant growth attributes, and biocontrol action under subtropical ecosystem
Source: PLoS One. 2021 Dec 16;16(12):e0261338. doi: 10.1371/journal.pone.0261338 (PMC8675670; doi:10.1371/journal.pone.0261338)
Supplement: S3 Fig — (DOCX) [file pone.0261338.s003.docx]

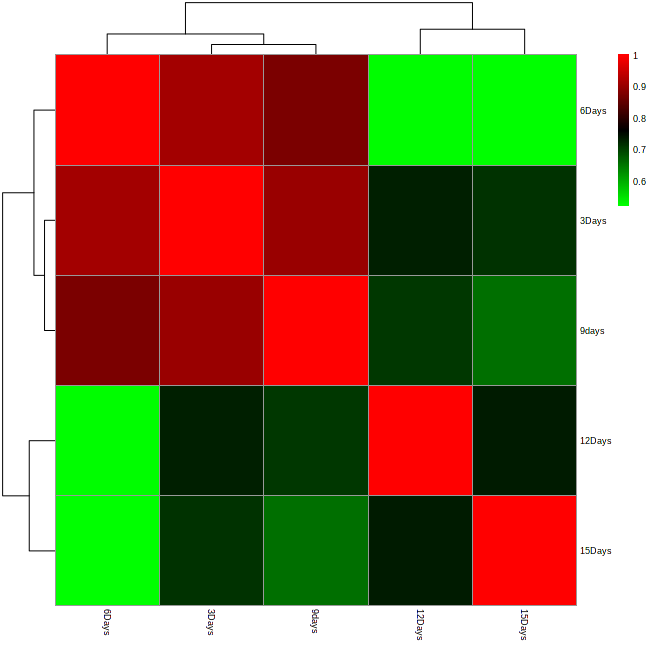
S3 Fig. Clustal Pearson correlation analysis of PBZ degradation under different treatment and different time intervals.
