# Supplementary material for: Exploration of Klebsiella pneumoniae M6 for paclobutrazol degradation, plant growth attributes, and biocontrol action under subtropical ecosystem
Source: PLoS One. 2021 Dec 16;16(12):e0261338. doi: 10.1371/journal.pone.0261338 (PMC8675670; doi:10.1371/journal.pone.0261338)
Supplement: S4 Fig — (DOCX) [file pone.0261338.s004.docx]

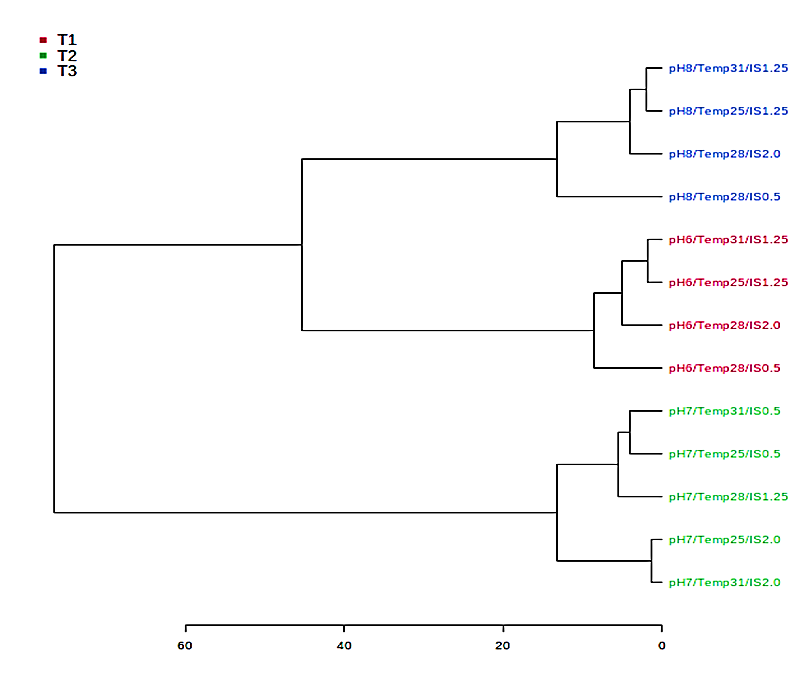


Cluster -1

Cluster -2

Cluster -3

Cluster -4

S4 Fig. Hierarchical cluster analysis of PBZ degradation under different treatments.
